# Timeliness metrics for screening and preventing TB in household contacts of pulmonary TB patients in Kenya

**DOI:** 10.5588/ijtldopen.23.0545

**Published:** 2024-01-01

**Authors:** D. Nair, P. Thekkur, I. Mbithi, M. Khogali, R. Zachariah, S. Dar Berger, S. Satyanarayana, A. M. V. Kumar, I. Kathure, J. Mwangi, A. F. Bochner, A. McClelland, J. M. Chakaya, A. D. Harries

**Affiliations:** ^1^ International Union Against Tuberculosis and Lung Disease (The Union), Paris, France;; ^2^ The Union South-East Asia Office, New Delhi, India;; ^3^ Respiratory Society of Kenya, Nairobi, Kenya;; ^4^ Institute of Public Health, College of Medicine and Health Sciences, University of the United Arab Emirates, Al Ain, United Arab Emirates;; ^5^ United Nations Children Fund, United Nations Development Programme, World Bank, WHO Special Programme for Research and Training in Tropical Diseases (TDR), Geneva, Switzerland;; ^6^ Yenepoya Medical College, Yenepoya (deemed to be University), Mangalore, India;; ^7^ Division of National TB, Leprosy and Lung Disease Programme, Ministry of Health, Nairobi,; ^8^ Department of Health, Kiambu County Government, Kiambu, Kenya;; ^9^ Resolve to Save Lives, New York City, NY, USA;; ^10^ Department of Medicine, Dermatology and Therapeutics, Kenyatta University School of Medicine, Nairobi, Kenya;; ^11^ Department of Clinical Research, Faculty of Infectious and Tropical Diseases, London School of Hygiene & Tropical Medicine, London, UK

**Keywords:** Kenya, HHC, household contact screening, TB preventive treatment, operational research, TPT

## Abstract

**BACKGROUND:**

The study assessed whether a “7-1-7” timeliness metric for screening and TB preventive therapy (TPT) could be implemented for household contacts (HHCs) of index patients with bacteriologically confirmed pulmonary TB under routine programmatic settings in Kenya.

**METHODS:**

A longitudinal cohort study conducted among index patients and their HHCs in 12 health facilities, Kiambu County, Kenya.

**RESULTS:**

Between January and June 2023, 95% of 508 index patients had their HHCs line-listed within 7 days of initiating anti-TB treatment (“First 7”). In 68% of 1,115 HHCs, screening outcomes were ascertained within 1 day of line-listing (“Next 1”). In 65% of 1,105 HHCs eligible for further evaluation, anti-TB treatment, TPT or a decision for no drugs was made within 7 days of screening (“Second 7”). Altogether, 62% of screened HHCs started TPT during the “7-1-7” period compared with 58% in a historical cohort. Main barriers to TPT uptake were HHCs not consulting clinicians, HHCs being unwilling to initiate TPT and drug shortages. Healthcare workers felt that a timeliness metric was valuable for streamlining HHC management and proposed “3-5-7” as a workable alternative.

**CONCLUSIONS:**

The national TB programme must generate awareness about TPT, ensure uninterrupted drug supplies and assess whether the “3-5-7” metric can be operationalised.

## RÉSUMÉ

**CONTEXTE:**

L'étude a évalué si une mesure de rapidité "7-1-7" pour le dépistage et le traitement préventif de la TB (TPT) pouvait être mise en œuvre pour les contacts familiaux des patients index atteints de TB pulmonaire confirmée bactériologiquement dans le cadre d'un programme de routine au Kenya.

**MÉTHODES:**

Étude de cohorte longitudinale menée auprès de patients index et de leurs contacts familiaux dans 12 établissements de santé du comté de Kiambu, au Kenya.

**RÉSULTATS:**

Entre janvier et juin 2023, 95% des 508 patients index ont eu leur centre de santé inscrit sur la liste dans les 7 jours suivant le début du traitement antituberculeux (« First 7 » ). Dans 68% des 1 115 centres de santé, les résultats du dépistage ont été vérifiés dans le jour suivant l'inscription sur la liste (« Next 1 »). Dans 65% des 1 105 centres de santé éligibles pour une évaluation plus approfondie, le traitement antituberculeux, le TPT ou la décision de ne pas prendre de médicaments a été prise dans les 7 jours suivant le dépistage (« Second 7 »). Au total, 62% des patients dépistés ont commencé un traitement antituberculeux au cours de la période « 7-1-7 », contre 58% dans une cohorte historique. Les principaux obstacles à l'adoption du TPT étaient les suivants : les centres de santé ne consultaient pas les cliniciens, les centres de santé n'étaient pas disposés à commencer le TPT et les pénuries de médicaments. Les professionnels de la santé ont estimé qu'une mesure de la rapidité d'exécution était utile pour rationaliser la gestion des centres de santé et ont proposé le « 3-5-7 » comme solution de rechange viable.

**CONCLUSION:**

Le programme national de lutte contre la TB doit sensibiliser au TPT, garantir un approvisionnement ininterrompu en médicaments et évaluer si la mesure « 3-5-7 » peut être mise en œuvre.

TB remains a global public health concern, with an estimated 10.6 million individuals developing TB in 2021.^1^ To achieve the End TB target of 90% reduction in TB incidence by 2035 (compared to 2015), optimal implementation of TB preventive interventions is necessary.^1,2^ The WHO recommends TB preventive therapy (TPT) under Pillar 1 of the End TB Strategy as TPT reduces TB incidence by limiting progression from TB infection to disease.^2^

Household contacts (HHC) of bacteriologically confirmed pulmonary TB patients are at high risk of exposure to TB infection and eventual disease. The WHO therefore recommends systematic screening of HHCs for TB and initiation of TPT after ruling out TB.^3,4^ Acknowledging the importance of TPT, world leaders committed to provide TPT in 2018 to at least 24 million HHC (target) by 2022.^5^ However, global implementation of TPT has been suboptimal, as only 2.2 million (9% of target) HHCs received TPT between 2018 and 2021.^6^ Challenges at different stages of HHC screening and management have led to this suboptimal coverage.^3,7,8^

Timely initiation of TPT is also crucial, as over 80% of child HHC who develop TB disease do so within the first 3 months of exposure.^9^ However, studies have reported that HHC screening and initiation of TPT among eligible contacts can take 2 months or longer.^10^ Thus, along with improving coverage of HHC screening and TPT, there is need to ensure prompt actions to harness TPT effectiveness.

To improve timeliness of HHC management and TPT initiation, we took inspiration from a new global target of “7-1-7” proposed to facilitate pandemic preparedness. In brief, this target is defined as follows: detection of an outbreak within 7 days of emergence, notification of the outbreak to public health authorities within 1 day of detection, and completion of early response actions within 7 days of notification.^11,12^ We adapted this metric for the process of HHC management as follows: 1) HHCs of the index TB patient are line-listed within 7 days of the index patient initiating anti-TB treatment (First 7); 2) line-listed HHCs are screened for symptoms suggestive of TB within the next 1 day (Next 1); 3) eligible HHCs are initiated on anti-TB treatment, TPT or a decision is taken to receive no drugs within 7 days of symptom screening (Second 7) (Table 1).

**Table 1.** “7-1-7” timeliness metrics in the context of outbreak preparedness and household contact management of bacteriologically confirmed TB patients.

Timeliness metrics	Outbreak preparedness	Household contact management
First-7	Detection of an outbreak within 7 days of its emergence	Household contacts of the index TB patient are line-listed within 7 days of the index patient initiating anti-TB treatment
Next-1	Notification of the outbreak to public health authorities within 1 day of detection	Line-listed household contacts are screened for symptoms suggestive of TB within the next 1 day of line-listing
Second-7	Completion of early response actions within 7 days of notification	Eligible household contacts are initiated on anti-TB treatment, TB preventive treatment or a decision is taken to receive no drugs within 7 days of symptom screening

This study assessed whether “7-1-7” was a workable timeliness metric for implementing screening and TPT initiation among HHCs of bacteriologically confirmed pulmonary TB patients initiated on treatment in public health facilities of Kiambu county, Kenya. The study was conducted in Kenya as it is a high TB burden country and the National TB Programme (NTP) was already implementing HHC screening and TPT. The specific objectives of the study were to 1) ascertain the proportions of TB patients and their HHCs who were screened, investigated and given appropriate interventions in each of the “7-1-7” stages, 2) compare proportions of HHCs screened and started on anti-TB treatment/TPT in the “7-1-7” cohort with a historical cohort to evaluate if overall TPT uptake improved, and 3) document on-field enablers and barriers in implementing the timeliness metric.

## METHODS

### Study design

This was a longitudinal cohort study.

### Setting

**Figure 1.**
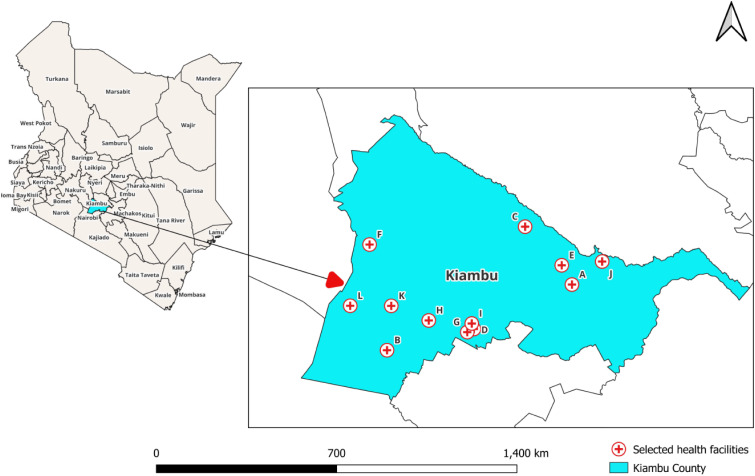
Map of Kenya showing the 12 facilities in Kiambu County where the “7-1-7” project was implemented. The selected health facilities had higher TB notifications due to the presence of TB clinics within each facility that had both diagnostic and treatment capabilities and accessibility by public transport. The socio-economic characteristics of the population attending these TB centres were not known to be different from the populations attending the other centres in Kiambu County, Nairobi.

The study was conducted in Kiambu County, adjacent to Nairobi, the capital city of Kenya. Of 144 TB health facilities in the county, 12 that recorded the highest number of TB registrations per quarter were selected (Figure 1).

Healthcare workers (HCWs) at the TB clinics in the health facilities comprised TB clinicians and community health volunteers (CHVs) who provided TB diagnosis and treatment as per national NTP standards.^13^ The CHVs line-listed HHCs of all bacteriologically confirmed pulmonary TB patients (index patients) according to the accepted generic HHC definition.^13^ They assessed each HHC using face-to-face interviews for symptoms suggestive of TB (cough, fever, weight loss, night sweats and haemoptysis) either through home visits or at the health facility.

The CHVs requested all HHCs to attend the health facilities for evaluation to determine the presence of active TB disease among those with chest symptoms and assess eligibility for TPT among those who were asymptomatic. HHCs in whom TB disease had been ruled out were initiated on TPT, providing there were no contra-indications. HHCs with TB disease were initiated on anti-TB treatment.

### Study population

All bacteriologically confirmed pulmonary TB patients registered for treatment in the 12 selected health facilities of Kiambu County and their line-listed HHCs were eligible for the study. The minimum calculated sample size was 450 index patients, assuming the achievement of the First 7 would be 80% with a relative precision of 5%, 95% confidence and 10% non-response. Assuming that 100 index patients would initiate treatment per month in the selected facilities, we included index patients from January to June 2023. A 3-month historical cohort (October–December 2022) enrolled prior to “7-1-7” implementation was included for comparison.

### Implementation of “7-1-7” metrics

Prior to implementation, the CHVs underwent a 2-day training on timeliness metrics and ways to integrate them into routine HHC screening and management. CHVs attempted to adhere to the “7-1-7” timeliness metrics and recorded the following data variables using a structured proforma: symptom screening outcome of HHCs already on anti-TB treatment or TPT, those with chest symptoms and those who were asymptomatic.

Based on the clinician’s evaluation, HHCs were classified as 1) diagnosed with TB and enrolled for anti-TB treatment, 2) eligible for TPT and started on TPT, or 3) decision made by either clinician or HHC not to receive anti-TB treatment or TPT. Dates were recorded as follows: date of treatment initiation in index patient; date of line-listing of HHC; date of HHC symptom screening; date of starting anti-TB treatment, TPT or a decision to receive no treatment. Data were captured on an EpiCollect5 (https://five.epicollect.net) mobile application. A study coordinator oversaw the work and attended to data quality check reports generated weekly by the Centre for Operational Research (COR), International Union Against Tuberculosis and Lung Disease (The Union), Paris, France. At the end of the “7-1-7” implementation, a review meeting was conducted and attended by staff from the health facilities, county-level NTP representatives and the investigators. Study findings were presented and insights regarding enablers, barriers, utility and feasibility of implementing timeliness metrics were obtained.

### Analysis and statistics

Data were analysed using STATA^®^ v16.0 (Stata Corp, College Station, TX, USA). Two analyses were conducted. First, numbers and proportions achieving each of the “7-1-7” components were analysed. Unadjusted binomial regression was used to assess associations of individual-level characteristics with achievement in each of the “7-1-7” components. Unadjusted relative risks with 95% confidence intervals (CIs) were used as measures of association. Second, aggregate data of index TB patients and HHCs screened and managed were compared between the “7-1-7” cohort and the historical cohort.

### Ethics

Ethics approval was obtained from the Ethics Advisory Group, The Union, Paris, France (EAG 04/2022 dated 28 June 2022) and from the Kenya Medical Research Institute, Nairobi, Kenya (KEMRI/RES/7/3/1 dated 23 October 2022). As the study was conducted under routine programme settings, a waiver of informed consent was granted by the ethics committees.

## RESULTS

### Implementation of the “7-1-7” metrics and characteristics of index patients and HHCs

**Figure 2.**
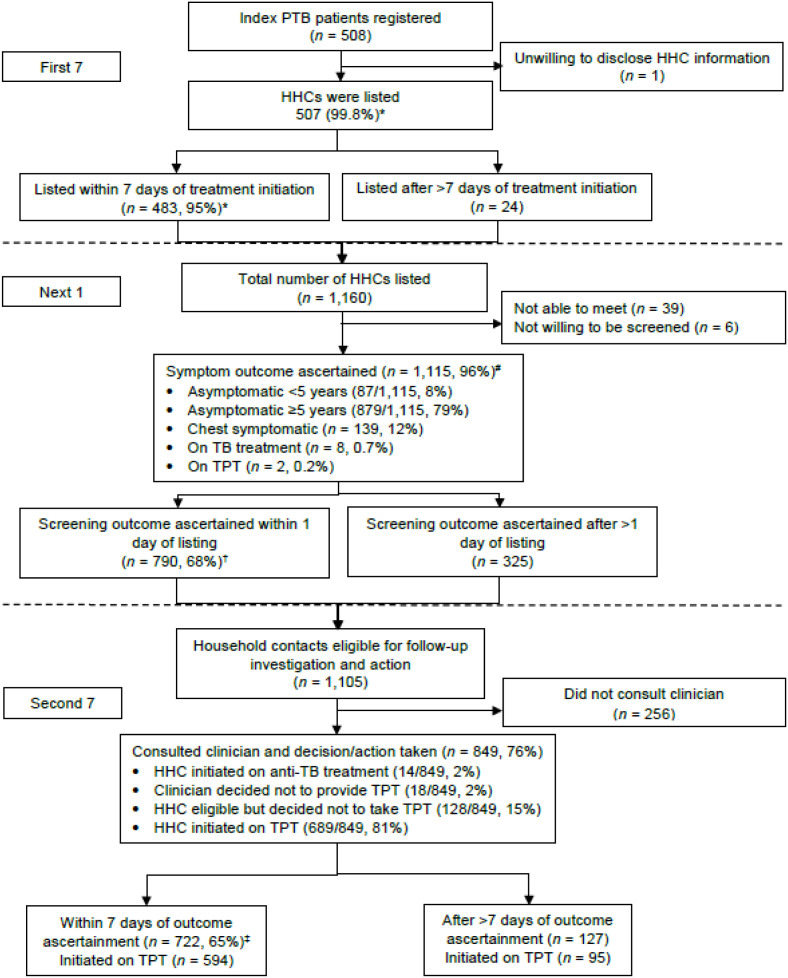
Overview of implementation of “7-1-7” among HHCs of patients with bacteriologically-confirmed PTB initiated on anti-TB treatment in Kiambu County, Kenya, January–June 2023. *Percentage calculated with total number of index patients as the denominator. ^†^Percentage calculated with total number of HHCs as denominator. ^‡^Percentage calculated with HHCs eligible for follow-up investigation and action as denominator. PTB = pulmonary TB; HHC = household contact; TPT = TB preventive treatment.

There were 508 index patients with 1,160 HHCs line-listed. HHC line-listing was achieved for 483/508 (95%) index patients within 7 days of treatment initiation; symptom screening outcomes were ascertained for 790/1,160 (68%) HHCs within 1 day after line-listing; and start of anti-TB treatment, TPT or a decision to receive neither was achieved in 722/1,105 (65%) HHCs within 7 days of symptom screening outcomes (Figure 2). Characteristics of 507 index patients in whom CHVs completed line-listing of their HHCs within the first 7 days are shown in Table 2. Line listing of HHCs of index patients aged <15 years was significantly more likely to be completed in 7 days compared to the 30–44-year age group. Characteristics of 1,160 HHCs who were line-listed, traced and had their screening outcomes ascertained by CHVs within the next 1 day are shown in Table 3. There was a significantly higher achievement in the “Next 1” among HHCs aged 15–29 and 45–59 years compared to those aged <15 years, partners of index patients compared to siblings and in most facilities compared to Facility L.

**Table 2.** Characteristics of index patients with bacteriologically confirmed PTB initiated on anti-TB treatment in relation to line-listing their HHCs and completing line-listing within the first 7 days in Kiambu County, Kenya, January–June 2023.

Characteristics of index PTB patients	Total *n*	Patients whose HHCs were line-listed *n* (%)*	Patients whose HHCs were line-listed within 7 days (“First 7”) *n* (%)*	Crude RR (95% CI)^†^	*P* value
Total	508	507 (99.8)	483 (95)	—	—
Age, years					
≤14	10	10 (100)	10 (100)	1.1 (1.0–1.1)	0.001
15–29	183	183 (100)	174 (95)	1.0 (0.9–1.1)	0.73
30–44	211	210 (99.5)	199 (94)	Reference	
45–59	82	82 (100)	79 (96)	1.1 (0.9–1.1)	0.44
≥60	22	22 (100)	21 (95)	1.0 (0.9–1.1)	0.81
Sex					
Male	359	358 (99.7)	339 (94)	Reference	
Female	149	149 (100)	144 (97)	1.0 (0.9–1.1)	0.25
Tuberculosis Unit					
A	51	51 (100)	48 (94)	1.1 (0.9–1.4)	0.25
B	11	11 (100)	11 (100)	1.2 (0.9–1.5)	0.08
C	21	20 (95)	20 (95)	1.2 (0.9–1.5)	0.23
D	141	141 (100)	139 (99)	1.2 (0.9–1.5)	0.11
E	17	17 (100)	14 (82)	Reference	
F	33	33 (100)	32 (97)	1.2 (0.9–1.5)	0.16
G	40	40 (100)	37 (93)	1.1 (0.9–1.4)	0.34
H	2	2 (100)	2 (100)	1.2 (0.9–1.5)	0.08
I	92	92 (100)	87 (95)	1.1 (0.9–1.4)	0.23
J	44	44 (100)	41 (93)	1.1 (0.8–1.4)	0.30
K	20	20 (100)	16 (80)	0.9 (0.7–1.3)	0.85
L	36	36 (100)	36 (100)	1.2 (0.9–1.5)	0.08

* Row percentage among total number of index PTB patients approached in each category.

^†^ The RR with 95% CIs for achieving the “First 7” milestone with total number of index PTB patients in each category as denominator. Failure to achieve the “First 7” includes individuals for whom HHCs were never listed and those for whom HHCs were listed after 7 days.

PTB = pulmonary TB; HHC = household contact; RR = relative risk; CI = confidence interval.

**Table 3.** Characteristics of HHCs of index patients with bacteriologically confirmed PTB who were traced, had their screening outcomes ascertained and completed outcome ascertainment within 1 day in Kiambu County, Kenya, January–June 2023.

Characteristics of HHCs	Total *n*	Traced *n* (%)*	Screening outcomes ascertained *n* (%)*	Screening outcomes ascertained within 1 day (“Next 1”) *n* (%)	Crude RR (95% CI)^†^	*P* value
Total	1,160	1.121 (97)	1,115 (96)	790 (68)		
HHC age, years						
≤14	370	360 (97)	358 (97)	233 (63)	Reference	
15–29	301	289 (96)	288 (96)	214 (71)	1.1 (1.0–1.3)	0.02
30–44	234	220 (94)	219 (94)	155 (66)	1.1 (0.9–1.2)	0.41
45–59	168	167 (99)	165 (98)	128 (76)	1.2 (1.1–1.4)	0.001
≥60	87	85 (98)	85 (98)	60 (69)	1.1 (0.9–1.3)	0.27
HHC sex						
Male	526	502 (95)	498 (95)	351 (67)	Reference	
Female	632	617 (98)	615 (97)	437 (69)	1.1 (0.9–1.1)	0.38
Others	2	2 (100)	2 (100)	2 (100)	—	
Relationship with index PTB patient						
Partner	146	143 (98)	142 (97)	109 (75)	1.2 (1.0–1.3)	0.03
Child	265	254 (96)	254 (96)	176 (66)	1.0 (0.9–1.2)	0.65
Parent	197	193 (98)	192 (97)	139 (71)	1.1 (0.9–1.2)	0.17
Siblings	248	241 (97)	240 (97)	160 (65)	Reference	
Others	304	290 (95)	287 (94)	206 (68)	1.1 (0.9–1.2)	0.42
Tuberculosis Unit						
A	96	94 (98)	94 (98)	68 (71)	1.8 (1.4–2.5)	<0.001
B	29	29 (100)	29 (100)	18 (62)	1.6 (1.1–2.4)	0.01
C	65	58 (89)	54 (83)	48 (74)	1.9 (1.4–2.6)	<0.001
D	288	281 (98)	281 (98)	218 (76)	2.0 (1.5–2.6)	<0.001
E	49	48 (98)	48 (98)	36 (73)	1.9 (1.4–2.6)	<0.001
F	99	90 (91)	89 (90)	47 (47)	1.2 (0.9–1.7)	0.19
G	92	85 (92)	85 (92)	71 (77)	2.0 (1.5–2.7)	<0.001
H	3	3 (100)	3 (100)	3 (100)	2.6 (2.0–3.4)	<0.001
I	152	150 (99)	150 (100)	118 (78)	2.0 (1.5–2.7)	<0.001
J	99	99 (100)	99 (100)	90 (91)	2.4 (1.8–3.1)	<0.001
K	91	91 (100)	90 (99)	36 (40)	1.0 (0.7–1.5)	0.84
L	97	93 (96)	93 (96)	37 (38)	Reference	
HHCs who were line-listed within the 7-day target of treatment initiation in the index patient						
Target achieved	1,104	1,066 (97)	1,1060 (96)	747 (68)	Reference	
Target not achieved	56	55 (98)	55 (98)	43 (77)	1.1 (0.9–1.3)	0.09

* Row percentage calculated among total number of household contacts in each category.

^†^ The RR with 95% CI for achieving the “next 1” milestone with total number of household contacts in each category as denominator. Not achieving the “next 1” includes those HHC in whom screening was never done and those in whom screening was done after 1 day.

HHC = household contact; PTB = pulmonary TB; RR = relative risk; CI = confidence interval.

Of 1,115 HHC screened, 8 were already on anti-TB treatment, 2 were already on TPT; therefore, 1,105 were eligible for further evaluation. Of 1,105 HHCs, 849 (76%) consulted a clinician, of whom 14 were diagnosed with TB and started on anti-TB treatment. Of the remaining 835 HHCs who did not have TB and were potentially eligible for TPT, 689 (83%) started TPT at any time, while 146 (17%) did not start TPT due to a decision made by either the clinician or HHC. Characteristics of 1,105 HHCs who were eligible for follow-up investigation and were started on anti-TB treatment, TPT or had a decision for no drugs and had these acted upon within the second 7 days are shown in Table 4. The “Second 7” phase was better achieved in most age groups compared to those aged <15 years, children of index patients compared to parents, and in most facilities compared to Facility C.

**Table 4.** Characteristics of HHCs of index patients with bacteriologically confirmed PTB with screening outcomes ascertained in whom decisions were made and action taken within 7 days of symptom screening in Kiambu County, Kenya, January–June 2023.

		Decisions and action taken					Decisions and action taken within 7 days (“Second 7”) *n* (%)*		
Characteristics of households contacts	Total *N*	Total *n* (%)*	ATT *n*	TPT *n*	Doctor did not prescribe TPT *n*	Eligible but HHC_s_ did not take TPT *N*		Crude RR (95% CI)^†^	*P* value
Total	1,105	849 (77)	14	689	18	128	722 (65)		
HHC age, years									
≤14	355	285 (80)	7	224	6	48	244 (69)	1.3 (1.1–1.7)	0.009
15–29	286	218 (76)	4	173	4	37	190 (66)	1.3 (1.0–1.6)	0.02
30–44	217	167 (77)	1	135	5	26	141 (65)	1.3 (1.0–1.6)	0.04
45–59	163	125 (77)	2	108	2	13	104 (64)	1.2 (0.9–1.6)	0.07
≥60	84	54 (64)	0	49	1	4	43 (51)	Reference	
HHC sex									
Male	491	370 (75)	10	291	9	60	316 (64)	Reference	0.49
Female	612	479 (78)	4	398	9	68	406 (66)	1.0 (0.9–1.1)	0.49
Others	2	0 (0)	0	0	0	0	0 (0)	—	
Relationship with index patient									
Partner	140	106 (76)	0	87	4	15	92 (66)	1.1 (0.9–1.3)	0.30
Child	249	217 (87)	6	174	3	34	185 (74)	1.2 (1.1–1.4)	0.003
Parent	191	144 (75)	2	128	1	13	115 (60)	Reference	
Siblings	238	172 (72)	3	143	3	23	148 (62)	1.0 (0.8–1.2)	0.68
Others	287	209 (73)	3	257	7	43	182 (63)	1.0 (0.9–1.2)	0.48
Symptom screening outcome									
Asymptomatic <5 years	87	63 (72)	1	44	2	16	53 (61)	Reference	
Asymptomatic ≥5 years	879	669 (76)	1	564	11	93	571 (65)	1.1 (0.9–1.3)	0.47
Chest symtomatic	139	117 (84)	12	81	5	19	98 (71)	1.2 (0.9–1.4)	0.15
Tuberculosis Unit									
A	94	72 (77)	2	50	0	20	60 (64)	1.4 (1.0–1.9)	0.04
B	29	29 (100)	1	23	0	5	20 (69)	1.5 (1.0–2.2)	0.03
C	53	34 (64)	1	29	1	3	24 (45)	Reference	
D	281	227 (81)	2	215	0	10	175 (62)	1.4 (1.0–1.9)	0.04
E	48	33 (69)	0	25	4	4	30 (63)	1.4 (0.9–1.9)	0.09
F	89	71 (80)	1	65	2	3	65 (73)	1.6 (1.2–2.2)	0.004
G	84	49 (58)	1	40	2	6	49 (58)	1.3 (0.9–1.8)	0.15
H	2	2 (100)	0	2	0	0	2 (100)	2.2 (1.6–2.9)	<0.001
I	150	102 (68)	3	72	4	23	91 (61)	1.3 (0.9–1.8)	0.08
J	98	64 (65)	1	48	0	15	55 (56)	1.2 (0.9–1.7)	0.22
K	89	88 (99)	0	65	2	21	83 (93)	2.1 (1.5–2.8)	<0.001
L	88	78 (89)	2	55	3	18	68 (77)	1.7 (1.2–2.3)	0.001
HHCs who were screened for symptoms within the 1-day target from the time of listing									
Target achieved	785	598 (76)	8	495	12	83	502 (64)	Reference	
Target not achieved	320	251 (78)	6	194	6	45	220 (69)	1.1 (0.9–1.2)	0.12

* Row percentage calculated among total number of HHCs whose screening outcomes were ascertained.

^†^ The RRs with 95% CIs for achieving the “Second 7” milestone with total number of household contacts whose screening outcomes were ascertained in each category as denominator. Failure to meet the “Second 7” criteria involves HHCs for whom decisions and/or actions were either never initiated or were implemented after a period of 7 days.

HHC = household contact; PTB = pulmonary TB; ATT = anti-TB treatment; TPT = TB preventive treatment; RR = relative risk; CI = confidence interval.

### Comparison of “7-1-7” and historical cohorts

The comparison of the two cohorts is shown in Table 5. The proportion of HHCs screened at any time was significantly higher in the historical cohort (99%) compared to the “7-1-7” cohort (96%). A higher proportion of HHCs were started on TPT during the “7-1-7” period (62%) compared to the historical cohort (58%), although this difference was not statistically significant. Before the implementation of the “7-1-7” programme, however, screening was mostly done by proxy, that is, the CHVs gathered information regarding symptoms among HHCs from the index patient. For the “7-1-7” programme, CHVs met each HHC individually at their home or at the health facility and asked them about their symptoms.

**Table 5.** Comparison of key characteristics and activities before and after implementation of “7-1-7” for household contact tracing of PTB patients initiated on treatment in Kiambu County, Kenya.

Characteristics and activities	Before “7-1-7”: October-December 2022* *n* (%)	During “7-1-7”: January-June 2023^†^ *n* (%)	*P* value
Index pulmonary TB patients registered	212	508	
HHCs line-listed	415	1,160	
HHCs screened at any time	413 (99)^‡^	1,115 (96)^‡^	<0.001
HHCs started on ATT at any time	15 (4)^§^	22 (2)^§^	0.07
HHCs started on TPT at any time	239 (58)^§^	689 (62)^§^	0.16

* Defined as index PTB patients only providing details of HHCs (proxy screening).

^†^ Defined as community health volunteers meeting with HHCs at their home or at the health facility.

^‡^ Denominator is the number of HHCs line-listed.

^§^ Denominator is the number of HHCs screened at any time. HHC = household contact; PTB = pulmonary TB; ATT = anti-TB treatment; TPT = TB preventive treatment.

### Enablers and barriers to implementing “7-1-7” metrics

First 7 enablers included the familiarity of TB clinicians and CHVs with the process of HHC listing by interviewing index patients as part of HHC management. Barriers included the reluctance of HHCs to disclose HHC information due to fear, stigma of TB and undisclosed polygamy. Wrong addresses and phone numbers provided by some patients made it difficult to meet and interview them. First 1 enablers included counselling of index TB patients by TB clinicians on the importance of contact screening, provision of financial support to CHVs for home visits and fixing appointments for interviewing HHCs via phone calls prior to home visits. Barriers included the need for multiple home visits if all HHCs were not available, lack of CHV interest in contact screening, HHC preference for screening by TB clinicians rather than CHVs and difficulty in contacting HHCs of migrant labourers who returned to rural areas for fear of disclosing TB disease status to employers.

Second 7 enablers included a recent mass media campaign by the NTP highlighting the importance of TPT and good engagement of index patients by CHVs which facilitated mobilisation of HHCs for evaluation. The major barrier was unwillingness of HHCs to visit health facilities for further evaluation – asymptomatic HHCs felt this was unnecessary and those with chest symptoms preferred over-the-counter medication. Reluctance to get evaluated was mainly due to fear, stigma and financial constraints. Shortage of TPT drugs and lack of confidence among HHCs about TPT effectiveness contributed to low TPT uptake.

### Utility of timeliness metrics

County-level programme managers and HCWs appreciated the timeliness metrics as they felt motivated to focus on HHC management in a systematic and timely manner. Concerted actions led to an increase in the number of HHCs put on TPT despite drug shortages.

**Figure 3.**
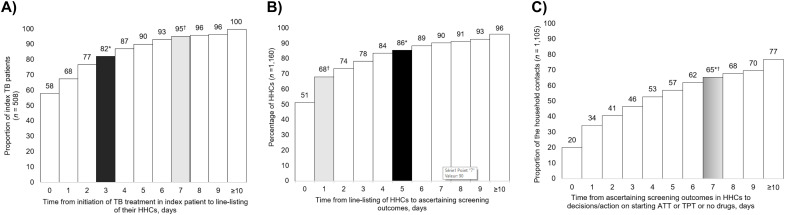
Daily cumulative achievement of outcomes for line-listing of HHCs of index patients with bacteriologically confirmed pulmonary TB, ascertainment of household contact screening outcomes and decisions/actions on anti-TB treatment, TPT or no drugs according to “7-1-7” or “3-5-7” timeliness metrics, Kiambu County, Kenya, January–June 2023. **A)** Time from initiation of TB treatment in index patient to line-listing of their HHCs; **B)** time from line-listing of HHCs to ascertaining screening outcomes; **C)** Time from ascertaining screening outcomes in HHCs to decisions/action on starting ATT or TPT or no drugs. *The “3-5-7” timeliness metric proposed by the community health volunteers (in black); ^†^the “7-1-7” timeliness metric (in grey). HHC = household contact; ATT = anti-TB treatment; TPT = TB preventive treatment.

CHVs suggested that a time metric of “3-5-7” would be more workable compared to “7-1-7”. On applying “3-5-7” metrics to this cohort, line-listing of HHC would be completed within 3 days among 82% of index patients, symptom screening outcomes ascertained within 5 days of listing in 89% of HHCs, and decisions and actions taken within 7 days of screening in 65% of HHCs (Figure 3).

## DISCUSSION

This is the first study to assess feasibility of implementing timeliness metrics for HHC management and TPT within the programmatic setting in Kenya. There were three key findings and implications for NTPs.

First, HCWs were able to list HHC in almost all index patients within 7 days of initiating treatment (95%), and they suggested that this activity could be completed within 3 days. This was because each facility had HCWs trained in NTP guidelines for HHC management. As seen in other studies, stigma and lack of awareness regarding TPT among index patients hindered line-listing efforts.^14–16^ However, counselling by HCWs helped to overcome this challenge. NTPs planning to roll out TPT for HHCs should specifically train HCWs on counselling index patients regarding HHC management.

Second, although almost all line-listed HHCs were screened for symptoms of TB, only 68% were screened within 1 day of listing. The availability of CHVs for in-field follow-up and screening of HHCs was pivotal in achieving high screening coverage. Better screening coverage was observed in the historical cohort period because index patients informed HCWs about symptoms of their HHCs (proxy screening), whereas during “7-1-7” each HHC was independently met and screened face-to-face in line with NTP guidelines. Similar to other studies, unavailability of HHCs during home visits, especially among migrants, hindered screening efforts.^17–19^ To overcome these challenges, CHVs had to make multiple visits, and this required more than 1 day for completing the activity. The NTPs should have dedicated staff for HHC management and should consider incentivising TB survivors or volunteers like TB Champions and TB Nanbans in India.^20^

Third, only three quarters of HHCs had actions/decisions taken after screening. In the remainder, many HHCs were unwilling to visit health facilities for further evaluation due to fear, stigma and financial constraints. Similar challenges have been reported from other settings.^16,19,21,22^ If these issues were addressed, the percentage of HHCs having actions/decisions taken within 7 days of screening could be improved from the current 65%. HCWs should focus on counselling HHCs on the need for further evaluation irrespective of symptoms. NTPs can also consider financial support to HHCs to cover travel costs to health facilities and to reimburse wage losses. Some HHCs also did not take up TPT due to unwillingness or shortage of TPT drugs. NTPs need to improve awareness of the benefits of TPT for HHCs, ensure uninterrupted drug supplies and consider switching to shorter TPT regimens.

The study had several strengths. It was conducted within the routine programmatic setting, data quality was assured through weekly checks, and the conduct and reporting of the study followed STROBE (Strengthening the Reporting of Observational studies in Epidemiology) guidelines.^23^ The main limitation was the lack of a concurrent control group for comparing the impact of introducing timeliness metrics. Comparisons with the historical cohort had the following limitations: 1) the time taken to complete the various steps of HHC management was unavailable; and 2) there were no TPT drug shortages during the historical cohort period. Furthermore, we were unable to conduct qualitative interviews among HHCs to understand their perspectives regarding timely HHC management.

The HCWs perceived timeliness metrics as workable and valuable for delivering TB preventive services as it motivated them and brought structure and promptness to the activity. Moving forwards, we need to assess whether the proposed “3-5-7” metric is achievable and can be scaled up. Although the process of HHC management was streamlined, there was no major improvement in TPT uptake as challenges related to visiting health facilities, HHC unwillingness to take TPT and drug shortages remained unaddressed. NTPs must take steps to improve awareness among HHCs regarding the importance of TPT and the process of screening and evaluation. At the same time, NTPs should ensure uninterrupted TPT drug stocks for improving TPT coverage.
